# Changes in Medication Use During Medicaid Continuous Enrollment and Unwinding

**DOI:** 10.1001/jamahealthforum.2025.5890

**Published:** 2026-01-02

**Authors:** Benjamin N. Rome, Jihye Han, Adrianna McIntyre, Aaron S. Kesselheim, Benjamin D. Sommers

**Affiliations:** 1 Program On Regulation, Therapeutics, And Law (PORTAL), Division of Pharmacoepidemiology and Pharmacoeconomics, Department of Medicine, Brigham and Women’s Hospital and Harvard Medical School, Boston, Massachusetts; 2 Department of Health Policy and Management, Harvard T.H. Chan School of Public Health, Boston, Massachusetts; 3 Department of Medicine, Brigham and Women’s Hospital and Harvard Medical School, Boston, Massachusetts

## Abstract

**Importance:**

During the COVID-19 pandemic, Medicaid enrollment increased because states suspended routine eligibility determinations. After this continuous enrollment provision ended in April 2023, millions of US individuals lost Medicaid coverage.

**Objective:**

To measure how the unwinding of Medicaid enrollment was associated with changes in patients’ use of health services, such as prescription medications.

**Design, Setting, and Participants:**

A cross-sectional study was carried out using interrupted time series analysis to compare changes in quarterly Medicaid enrollment and prescription medication use from 2018, quarter (Q) 1 through 2024, Q1. Data were analyzed from November 2024 to February 2025.

**Exposures:**

The onset of continuous enrollment provision (2020, Q2) and unwinding (2023, Q2).

**Main Outcomes and Measures:**

The outcomes were quarterly state Medicaid enrollment and estimated number of reimbursed prescriptions. Log-transformed linear regression models were used to compare changes in state enrollment and prescriptions after continuous enrollment and unwinding, overall and stratified by states with different net enrollment changes and policies to protect patients during unwinding. Subsets of medications for certain chronic conditions and formulations primarily used by children were analyzed.

**Results:**

In the quarter before the COVID-19 pandemic (2019, Q4), Medicaid enrollment was 71.4 million, and there were about 183.2 million prescriptions reimbursed by Medicaid programs. This included 59.1 million (32.3%) prescriptions treating chronic diseases, 30.3 million (16.5%) for acute conditions, and 15.0 million (8.2%) for other specified conditions. In 2023, Q2, enrollment peaked at 93.9 million (31.4% increase from baseline), and the number of prescriptions peaked at 212.6 million (16.1% increase from baseline). Enrollment increased by 2.42% (95% CI, 2.15%-2.70%) per quarter during continuous enrollment and decreased by 4.92% (95% CI, −6.12% to −3.70%) per quarter during unwinding. Concurrently, the number of prescriptions increased by 1.85% (95% CI, 1.21%-2.50%) per quarter and then decreased by 3.94% (95% CI, −5.73% to −2.11%) per quarter. Trends were similar for chronic disease medications and pediatric-specific formulations. States with the highest disenrollment during unwinding had the largest decreases in chronic disease medication use; states that implemented more protective policies had smaller decreases in enrollment and insignificant decreases in chronic medication use.

**Conclusions and Relevance:**

This cross-sectional study found that changes in Medicaid medication use during the COVID-19 pandemic continuous enrollment period and after unwinding were smaller than corresponding changes in enrollment. Unwinding had measurable impacts on patient access to prescription medications, but states that implemented protective policies were able to mitigate these changes.

## Key Points

**Question:**

How did the use of prescription medications change during periods of Medicaid continuous enrollment during the COVID-19 pandemic and the subsequent unwinding of coverage once the continuous enrollment provision ended in April 2023?

**Findings:**

In this cross-sectional study including all Medicaid enrollees from 2018 through the first quarter of 2024, state Medicaid enrollment increased by 2.42% per quarter during continuous enrollment and decreased by 4.92% per quarter during unwinding; concurrently, the number of prescriptions increased by 1.85% per quarter and then decreased by 3.94% per quarter.

**Meaning:**

Expansion and unwinding of Medicaid coverage had measurable impacts on patient access to prescription medications, and these changes were affected by state policies.

## Teaser

This cross-sectional study analyzes how the unwinding of Medicaid enrollment is associated with changes in patients’ use of health services, such as prescription medications.

## Introduction

During the COVID-19 public health emergency, enrollment in Medicaid and the Children’s Health Insurance Program (CHIP) grew rapidly, in large part due to the continuous enrollment provision included in the Families First Coronavirus Response Act of 2022.^1^ Under this provision, states received extra federal funding if they halted regular redeterminations of beneficiary eligibility and instead allowed all beneficiaries (except those moving out of state or who actively withdrew from the program) to maintain continuous Medicaid coverage. This provision ended in March 2023, after which states resumed eligibility redeterminations. Subsequently, more than 20 million beneficiaries lost Medicaid coverage, a period known as the unwinding of the continuous enrollment provision.^2,3^

Increased Medicaid enrollment during the COVID-19 pandemic improved access to care for vulnerable patients who frequently move in and out of the Medicaid program. This can occur because of intermittent ineligibility due to fluctuations in income or because of failure to comply with the necessary administrative steps to maintain continuous enrollment. By contrast, the unwinding process may have had adverse impacts on access to essential health care. However, survey data suggested that many patients who remained enrolled in Medicaid due to the continuous enrollment provision did not realize they had Medicaid coverage, which might have dampened the positive impact of these coverage changes on patient outcomes.^4^

An important measure of how the continuous enrollment provision—and its subsequent unwinding—affected clinical care is whether changes in Medicaid enrollment were correlated with changes in the use of essential health care services, such as prescription medications. Prescription medications are the mainstay of treatment for many chronic diseases, and adherence to medications is closely linked with insurance coverage. Cost-related nonadherence to essential medicines is associated with increases in morbidity and mortality.^5,6,7^ All state Medicaid programs cover medications with little or no cost-sharing requirement for beneficiaries. This level of cost-sharing is associated with improved adherence for essential medications,^7^ and insurance discontinuities—such as those caused by the unwinding of the continuous enrollment provision—are associated with decreased use of medications.^8^

Understanding the effects of the continuous coverage provision and subsequent unwinding on health care access is particularly important now because Congress recently enacted several changes to the Medicaid program that would increase administrative hurdles to remain continuously enrolled in the program and shift additional program costs to states.^9^ We therefore assessed how changes in Medicaid enrollment during and after the COVID-19 public health emergency corresponded with changes in prescription medications used by Medicaid patients, and explored how patterns varied by drug class, patient population, and state policy environment.

## Methods

### Data Sources

We measured the use of prescription medications using publicly reported Medicaid State Drug Utilization Data, which includes quarterly sums of the number of prescriptions and units (eg, tablets, milliliters) for each drug reimbursed by Medicaid program in all 50 states and Washington, DC.^10^ These data have been previously used to measure trends in the use and spending of medications.^11,12,13,14,15^ Data were obtained in October 2024 from the Centers for Medicaid & Medicaid Services (CMS), covering the study period from the first quarter of 2018 through the first quarter of 2024.

We identified drugs that treat certain chronic and acute conditions (described herein) using World Health Organization Anatomical Therapeutic Chemical (WHO-ATC) classification codes and identified drugs primarily used by pediatric populations using 2020 prescription claims data from the Transformed Medicaid Information System Analytic Files (TAF), which compiles individual pharmacy claims reimbursed by each state Medicaid program (eMethods, eFigure 1 in Supplement 1).

Monthly overall and pediatric (18 years and younger) state Medicaid and the Children’s Health Insurance Program (CHIP) enrollment were obtained from public CMS data (eMethods in Supplement 1).^16^ The Mass General Brigham institutional review board deemed this study exempt from review and written informed consent because it involved secondary use of deidentified data. The study was reported in accordance with Strengthening the Reporting of Observational Studies in Epidemiology (STROBE) reporting guidelines.

### Study Design and Exposure

Interrupted time series models were used to measure changes in quarterly Medicaid enrollment and prescription drug use after the Medicaid continuous enrollment provision went into effect (March 18, 2020) and ended (March 31, 2023).^17,18^ Based on these dates, we divided the study period into 3 time periods: before (2018, quarter [Q] 1 to 2020, Q1), during (2020, Q2 to 2023, Q1), and after (2023, Q2 to 2024, Q1) the continuous enrollment provision. Because some states did not begin processing eligibility redeterminations until summer 2023, in a sensitivity analysis we treated the unwinding period as beginning in 2023, Q3. In a second sensitivity analysis, we excluded 9 states that adopted Medicaid coverage expansion during our study period,^19^ because expansion might have altered enrollment trends and Medicaid population characteristics in these states. In a different sensitivity analysis, we included states that expanded Medicaid but adjusted for an indicator of whether states had adopted Medicaid expansion in each quarter^19^ as well as mean quarterly unemployment rates to ensure that changes between states were not due to different economic circumstances.^20^

### Outcomes

The primary outcome was the number of prescriptions reimbursed by state Medicaid and CHIP programs in each quarter. We removed 0.005% of state-drug quarters that were outliers likely resulting from erroneous state-reported data (eMethods in Supplement 1). In a sensitivity analysis, we measured changes in the mean number of prescriptions per Medicaid enrollee to understand how utilization changed among those enrolled in the program.

Secondary outcomes included the number of prescriptions for certain categories of drugs used to treat prevalent chronic or acute conditions in the Medicaid population (eTable 1 in Supplement 1).^21^ Medications for chronic conditions included those treating cardiovascular disease, diabetes, cancer, HIV, and psychiatric diseases (including antidepressants, antipsychotics, and stimulants). Medications for acute conditions included anti-infectives (except for antiviral medications for HIV, hepatitis C, and COVID-19), and cold and allergy medications. Other medication classes of interest that were not included in the chronic or acute categories included medications for obstructive pulmonary disease (eg, inhalers, which can be used for acute or chronic lung conditions), hormonal contraceptives, and antiviral medications for hepatitis C.

### Statistical Analysis

First, we plotted relative changes in national enrollment and medication use in each quarter, compared with the final quarter of 2019. Next, we created a series of segmented regression models to measure changes in Medicaid/CHIP enrollment and medication use during and after the continuous enrollment provision (eMethods in Supplement 1). Separate models were created for total and pediatric enrollment; total medication use; use of medications for selected chronic, acute, and other conditions; and use of pediatric-specific medications. The outcome for each model was the log-transformed count of enrolled beneficiaries or number of prescriptions; the log-transformed outcomes allowed us to measure relative changes. We used postestimation *t* tests to compare model coefficients for enrollment and medication use.

Since the COVID-19 pandemic was associated with abrupt changes in health care access and use,^22^ we allowed for level changes in each outcome after the start of the continuous enrollment provision. We did not include a level change at the start of unwinding because the transition to unwinding was more gradual with state variation in timing of resuming eligibility redetermination. To account for the time-invariant features of each state, including different Medicaid program sizes and patient populations, models included state fixed effects. To better approximate national trends, models were weighted by state Medicaid enrollment in the first quarter of the study (2018, Q1); unweighted results were reported in a sensitivity analysis. Standard errors were adjusted for clustering within states.

To assess for differences based on how states handled disenrollment after the continuous enrollment provision expired, we measured changes in enrollment and medication use with 2 sets of stratified models. First, we stratified states into quartiles based on the relative net change in Medicaid enrollment from March 2023 to April 2024 (eTable 2 in Supplement 1). Second, we stratified states based on a previously published^2^ measurement of how many policies states adopted to protect patient access during the unwinding period, such as spreading terminations over a year instead of shorter periods and using multiple data sources to reduce beneficiary reporting burden (eTable 3 in Supplement 1). We tested for differences in model coefficients between stratified state groups using post-estimation *t* tests.

All analyses were performed at the state-calendar quarter level using R statistical software (version 4.4.1., R Foundation). Statistical tests were 2-sided and deemed significant at *P* < .05. Data were analyzed from November 2024 to February 2025.

## Results

In the quarter before the COVID-19 pandemic (2019, Q4), Medicaid enrollment was 71.4 million, and there were about 183.2 million prescriptions reimbursed by Medicaid programs (Table 1). This included 59.1 million (32.3%) prescriptions treating chronic diseases, 30.3 million (16.5%) for acute conditions, and 15.0 million (8.2%) for other specified conditions. As a proportion of these baseline levels, enrollment increased more than the change in the number of prescriptions filled by Medicaid patients during the COVID-19 pandemic (Figure). In 2023, Q2, enrollment peaked at 93.9 million (31.4% increase from baseline), and the number of prescriptions peaked at 212.6 million (16.1% increase from baseline). Prescriptions for chronic diseases closely tracked enrollment; by contrast, the number of prescriptions for acute conditions dropped at the start of the COVID-19 pandemic and then increased and peaked only slightly above the prepandemic baseline. Trends for individual classes of medications are shown in eFigure 2 in Supplement 1, and for pediatric enrollment and medications are in eFigure 3 in Supplement 1.

**Table 1.** Number of Enrollees and Estimated Prescriptions Before, During, and After the Medicaid Continuous Enrollment Provision^a^

Outcome	No.		
	Before continuous enrollment provision (2019, Q4)	End of continuous enrollment provision (2023, Q1)	End of study period (2024, Q1)
Enrollment	71 425 969	93 729 657	84 093 469
Estimated prescriptions	183 169 408	212 622 664	193 835 145
Chronic diseases	59 131 258	74 394 726	66 096 127
Cancer	402 728	380 694	348 106
Cardiovascular	26 490 847	32 589 470	28 269 691
Diabetes	7 704 028	10 564 877	9 550 413
HIV	624 220	684 982	643 170
Psychiatric^b^	23 909 435	30 174 703	27 284 747
Acute conditions	30 303 355	32 945 426	30 752 423
Anti-infectives	17 984 089	20 290 919	18 547 589
Cold and allergy	12 319 266	12 654 507	12 204 834
Other drug classes			
Asthma and COPD	11 908 324	12 270 262	11 146 616
Hepatitis C antivirals	40 001	31 344	29 128
Contraceptives	3 025 634	3 616 846	3 037 635
Other^c^	78 760 836	89 364 060	82 773 216
Pediatric			
Enrollment	35 417 089	42 113 088	38 016 645
Medication use	16 786 896	14 915 314	13 447 504

Abbreviations: COPD, chronic obstructive pulmonary disease; Q, quarter.

^a^ The Table shows national sums for Medicaid enrollment and the estimated number of medications (overall and for selected subsets of drugs). Data are from 3 calendar quarters during the study period: before the continuous enrollment provision (2019, Q4), at the end of the continuous enrollment period at peak enrollment (2023, Q1), and the final time point in the study during unwinding (2024, Q1).

^b^ Includes antidepressants, antipsychotics, and stimulants.

^c^ Includes all medications not in one of the specified categories.

**Figure.**
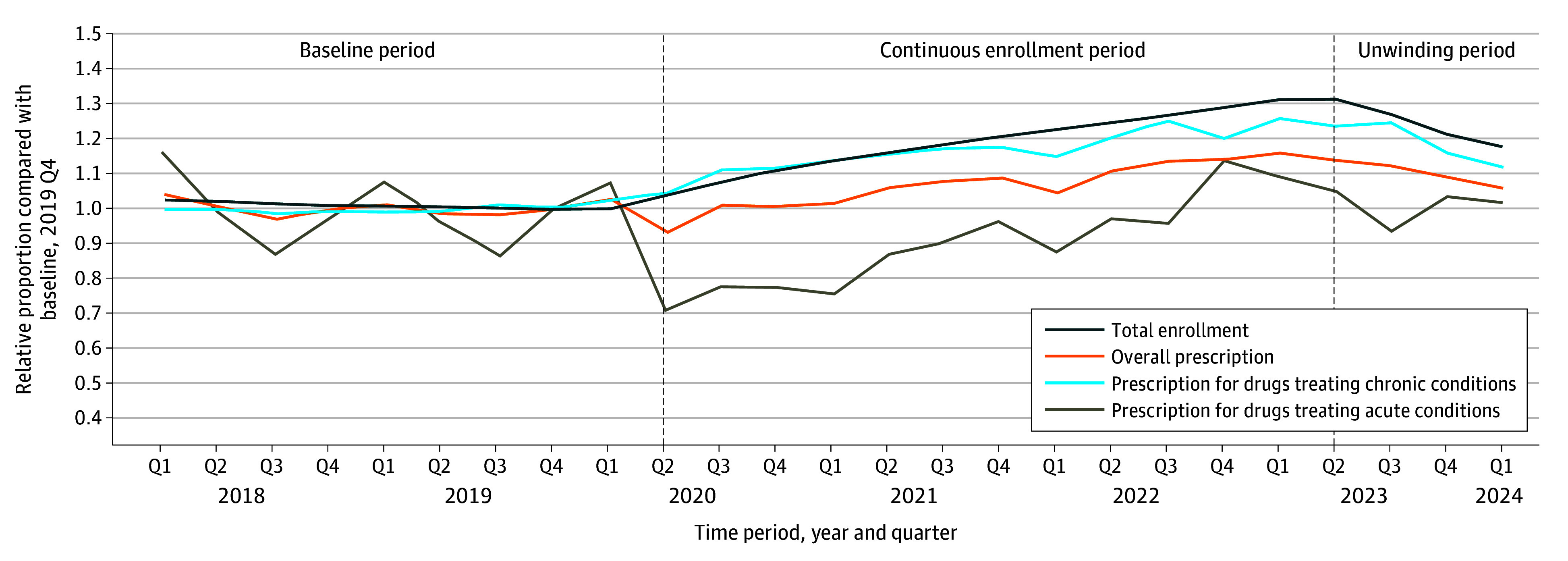
Changes in Medicaid Enrollment and Medication Use, 2018 to 2024 Each line shows relative changes in each quarter (Q) in national Medicaid enrollment, the total number of prescriptions reimbursed by Medicaid, and the number of prescriptions treating certain chronic and acute conditions. All values are shown as relative proportions to the baseline quarter (Q4 of 2019).

In time series models, state Medicaid enrollment decreased by 0.33% per quarter (95% CI, −0.54% to −0.12%) during the prepandemic period; the baseline trend in medication use was similar (*P* = .10) and did not differ from zero (Table 2). At the start of the continuous enrollment provision in March 2020, enrollment increased by 3.93% (95% CI, 2.83%-5.05%), whereas overall medication use decreased by 5.40% (95% CI, −8.12% to −2.59%; *P* < .001). Use of chronic medications increased similarly to enrollment (4.87%; 95% CI, 2.71%-7.08%; *P* = .24), while medications for acute conditions fell sharply (28.56% decrease; 95% CI, −31.50% to −25.49%; *P* < .001). There was a notable large abrupt 14.58% increase (95% CI, 8.51%-20.99%) in the use of contraceptives, and 23.98% decline (95% CI, −28.87% to −18.75%) in the use of hepatitis C antiviral medications.

**Table 2.** Changes in Medicaid Enrollment and Prescription Drug Use During the Medicaid Continuous Enrollment Provision and Unwinding

Outcome	% Per quarter (95% CI)^a^			
	Baseline trend, before the continuous enrollment provision	Level change, start of continuous enrollment provision	Trend change, during the continuous enrollment provision	Trend change, during unwinding
Enrollment	−0.33 (−0.54 to −0.12)	3.93 (2.83 to 5.05)	2.42 (2.15 to 2.70)	−4.92 (−6.12 to −3.70)
Estimated No. of prescriptions	−0.11 (−0.55 to 0.33)	−5.40 (−8.12 to −2.59)^b^	1.85 (1.21 to 2.50)^b^	−3.94 (−5.73 to −2.11)^b^
Chronic diseases	0.26 (−0.18 to 0.71)^b^	4.87 (2.71 to 7.08)	1.11 (0.64 to 1.59)^b^	−3.80 (−5.38 to −2.20)^b^
Cancer	0.50 (−0.94 to 1.97)	1.12 (−5.14 to 7.79)	−1.16 (−2.55 to 0.24)^b^	−3.10 (−5.87 to −0.25)
Cardiovascular	0.22 (−0.31 to 0.76)^b^	6.92 (4.19 to 9.72)^b^	0.90 (0.33 to 1.46)^b^	−4.17 (−5.59 to −2.72)
Diabetes	0.50 (0.03 to 0.98)^b^	5.87 (3.39 to 8.40)	1.59 (1.11 to 2.08)^b^	−3.98 (−6.05 to −1.86)^b^
HIV	−2.06 (−2.90 to −1.22)^b^	−0.32 (−2.63 to 2.04)^b^	3.56 (2.46 to 4.67)^b^	−3.19 (−4.93 to −1.42)^b^
Psychiatric^c^	0.37 (−0.00 to 0.74)	3.08 (1.11 to 5.08)	1.08 (0.61 to 1.55)	−3.41 (−5.05 to −1.75)
Acute conditions	−0.35 (−0.74 to 0.04)	−28.56 (−31.50 to −25.49)^b^	4.03 (3.11 to 4.95)^b^	−5.73 (−8.35 to −3.03)
Anti-infectives	−0.41 (−0.78 to −0.04)	−36.64 (−39.89 to −33.22)^b^	5.39 (4.39 to 6.40)^b^	−7.12 (−9.72 to −4.45)^b^
Cold and allergy	−0.19 (−0.70 to 0.33)	−15.89 (−20.33 to −11.21)^b^	2.08 (1.16 to 3.02)	−3.92 (−6.56 to −1.21)^b^
Other drugs				
Asthma and COPD	0.30 (−0.13 to 0.73)^b^	−10.40 (−14.55 to −6.05)^b^	1.05 (0.63 to 1.47)^b^	−3.27 (−4.75 to −1.77)^b^
Hepatitis C antivirals	0.77 (−2.52 to 4.17)	−23.98 (−28.87 to −18.75)^d^,^b^	−0.14 (−4.01 to 3.89)^b^	−3.21 (−8.30 to 2.17)
Contraceptives	−0.25 (−0.80 to 0.30)	14.58 (8.51 to 20.99)^b^	0.97 (0.14 to 1.81)^b^	−6.34 (−9.05 to −3.55)^b^
Other	−0.37 (−0.90 to 0.16)	−3.16 (−5.98 to −0.25)	1.79 (1.09 to 2.50)^d^	−3.33 (−4.94 to −1.70)^d^
Pediatric				
Enrollment	−0.42 (−0.60 to −0.24)	2.69 (1.71 to 3.68)	1.66 (1.40 to 1.91)	−3.47 (−4.60 to −2.32)
Medication use	0.19 (−0.14 to 0.52)^b^	−43.43 (−47.84 to −38.65)^b^	4.02 (3.40 to 4.66)^b^	−7.21 (−9.30 to −5.06)^b^

Abbreviations: COPD, chronic obstructive pulmonary disease; Q, quarter.

^a^ The baseline period was 2018, Q1 to 2020, Q1, the continuous enrollment period was 2020, Q2, to 2023, Q1, and the unwinding period was 2023, Q2, to 2024, Q1.

^b^ *P* < .05 for the postestimation coefficient test, compared with the coefficient for enrollment.

^c^ Includes antidepressants, antipsychotics, and stimulants.

During the continuous enrollment period, Medicaid enrollment increased by 2.42% (95% CI, 2.15%-2.70%) per quarter, while overall medication use increased by 1.85% (95% CI, 1.21%-2.50%) per quarter (*P* = .003). The trend in the quarterly number of estimated prescriptions increased for all classes of medications examined, except for medications treating cancer and hepatitis C virus.

During unwinding, Medicaid enrollment decreased by 4.92% (95% CI, −6.12% to −3.70%) per quarter; there was a smaller decrease in the number of prescriptions (−3.94%; 95% CI, −5.73% to −2.11%) per quarter (*P* = .005). Reductions in prescriptions for chronic diseases (−3.80%; 95% CI, −5.38% to −2.20%) were generally smaller than reductions in enrollment (*P* = .003); quarterly trends ranged from −3.10% per quarter for cancer to −4.17% per quarter for cardiovascular disease. The decrease in prescriptions for acute conditions (−5.73%; 95% CI, −8.35% to −3.03%) was similar to enrollment (*P* = .05), although there was a larger decrease for anti-infective medications (−7.12%; 95% CI, −9.72% to −4.45%; *P* < .001).

In contrast to overall trends, changes in the number of pediatric-specific medications outpaced changes in pediatric enrollment. During continuous enrollment, pediatric enrollment increased by 1.66% (95% CI, 1.40%-1.91%) per quarter, whereas pediatric-specific medication use increased by 4.02% (95% CI, 3.40%-4.66%) per quarter (*P* < .001); during unwinding, pediatric enrollment decreased by 3.47% (95% CI, −4.60% to −2.32%) per quarter and medication use decreased by 7.21% (95% CI, −9.30% to −5.06%) per quarter (*P* < .001).

Results were similar in sensitivity analyses that included a 1-quarter lag at the end of the continuous enrollment provision (eTable 4 in Supplement 1), when results were not weighted by baseline state Medicaid enrollment (eTable 5 in Supplement 1), excluding the 9 states that expanded Medicaid during the study period (eTable 6 in Supplement 1), and controlling for state unemployment rates and Medicaid expansion status (eTable 7 in Supplement 1). In general, the number of prescriptions per Medicaid beneficiary decreased during the continuous enrollment period and increased during the unwinding period (eTable 8 in Supplement 1).

In stratified analyses, the quartile of states with the largest declines in enrollment during unwinding experienced a 9.16% (95% CI, −10.45% to −7.85%) per quarter decrease in enrollment and a 7.16% (95% CI, −8.07% to −6.24%) decrease in the estimated use of chronic disease medications (Table 3). In the quartile of states with the smallest net enrollment declines, enrollment decreased by 2.60% (95% CI, −3.09% to −2.11%) and the estimated number of chronic disease medication prescriptions did not significantly change compared with the COVID-19 pandemic trend. Similarly, states that imposed 0 or 1 coverage-promoting policies during the unwinding period had a 7.83% (95% CI, −9.99% to −5.61%) per quarter decrease in enrollment and a 6.17% (95% CI, −7.68% to −4.64%) per quarter decrease in chronic disease medications, whereas states with 4 coverage-promoting policies had a smaller decrease in enrollment (3.41%; 95% CI, −4.48% to −2.33%) and no change in the number of chronic disease medications. Stratified results were similar when excluding 9 states that expanded Medicaid during the study period (eTables 9-10 in Supplement 1).

**Table 3.** Changes in Medicaid Enrollment and Medication Use, Stratified by State Characteristics

Outcome	% Per quarter (95% CI)^a^			
	Baseline trend, before the continuous enrollment provision	Level change, start of continuous enrollment provision	Trend change, during the continuous enrollment provision	Trend change, during unwinding
**Quartiles by net state enrollment change from March 2023 to April 2024^b^**				
Enrollment				
First quartile	−0.56 (−0.75 to −0.37)	4.56 (3.46 to 5.68)	3.18 (2.72 to 3.65)	−9.16 (−10.45 to −7.85)
Second quartile	−0.35 (−0.56 to −0.14)	3.94 (2.95 to 4.95)	2.37 (2.10 to 2.63)	−5.18 (−5.63 to −4.73)^c^
Third quartile	−0.37 (−0.80 to 0.06)	4.68 (3.33 to 6.04)	2.72 (2.00 to 3.44)	−4.76 (−5.56 to −3.95)^c^
Fourth quartile	−0.18 (−0.75 to 0.39)	3.26 (1.04 to 5.53)	1.95 (1.46 to 2.44)^c^	−2.60 (−3.09 to −2.11)^c^
No. of prescriptions for chronic disease medications				
First quartile	0.55 (−0.31 to 1.42)	3.55 (−1.43 to 8.78)	1.34 (0.52 to 2.16)	−7.16 (−8.07 to −6.24)
Second quartile	0.35 (0.01 to 0.69)	5.89 (4.19 to 7.63)	0.95 (0.56 to 1.33)	−4.35 (−4.96 to −3.74)
Third quartile	0.55 (−0.36 to 1.48)	3.86 (−1.34 to 9.33)	1.57 (0.69 to 2.47)	−4.73 (−5.57 to −3.88)^c^
Fourth quartile	−0.08 (−1.02 to 0.88)	5.41 (0.70 to 10.34)	0.88 (−0.14 to 1.91)	−1.03 (−2.35 to 0.32)^c^
**No. of coverage-promoting policies**				
Enrollment				
0-1 Policy	−0.55 (−0.78 to −0.31)	4.07 (2.63 to 5.54)	3.09 (2.57 to 3.61)	−7.83 (−9.99 to −5.61)
2 Policies	0.11 (−0.69 to 0.92)	4.07 (2.50 to 5.67)	1.94 (1.23 to 2.64)^c^	−4.65 (−6.21 to −3.06)^c^
3 Policies	−0.34 (−0.56 to −0.11)	4.28 (3.54 to 5.03)	2.49 (1.97 to 3.02)^c^	−4.87 (−6.09 to −3.64)^c^
4 Policies	−0.44 (−0.70 to −0.18)	3.45 (0.78 to 6.20)	2.25 (2.00 to 2.49)^c^	−3.41 (−4.48 to −2.33)^c^
No. of prescriptions for chronic disease medications				
0-1 Policy	0.01 (−0.31 to 0.33)	2.83 (−1.92 to 7.81)	1.51 (0.81 to 2.22)	−6.17 (−7.68 to −4.64)
2 Policies	0.67 (−0.99 to 2.36)	9.59 (4.13 to 15.33)	0.82 (−0.77 to 2.43)	−4.37 (−6.38 to −2.33)
3 Policies	0.28 (−0.23 to 0.79)	5.20 (2.85 to 7.61)	1.18 (0.68 to 1.68)	−3.35 (−4.71 to −1.98)^c^
4 Policies	0.18 (−0.65 to 1.01)	3.34 (0.08 to 6.72)	0.98 (0.09 to 1.87)	−2.46 (−5.39 to 0.56)^c^

^a^ The baseline period was 2018, quarter 1 (Q1), to 2020, Q1, the continuous enrollment period was 2020, Q2, to 2023, Q1, and the unwinding period was 2023, Q2, to 2024, Q1.

^b^ First quartile includes the quartile of states with the largest negative net changes (eg, largest decrease) in net Medicaid enrollment from March 2023 to April 2024. Fourth quartile includes states with the smallest decreases (or increases) in enrollment.

^c^ *P* < .05 for the postestimation coefficient test, compared with the coefficient for the first quartile or 0 to 1 policy group.

## Discussion

Use of prescription medications in Medicaid increased concurrently with expanded enrollment during the continuous Medicaid coverage period of the COVID-19 pandemic and decreased as enrollment declined after this provision ended in 2023. The changes in Medicaid enrollment during the COVID-19 pandemic thus had measurable impacts on access to essential health care services, such as prescription medications to treat chronic conditions like diabetes, cardiovascular disease, and cancer.

The results from this study add to a growing body of evidence pointing to the negative impacts of Medicaid unwinding on patients’ access to prescription drugs. Two previous studies^23,24^ found that patients in states with the greatest enrollment decreases during unwinding had more interruptions in chronic medications used to treat opioid use disorder and several pediatric neurologic and psychiatric conditions, compared with states less affected by unwinding. Our study showed that the unwinding period was associated with substantial reductions in medication use across a broader range of medications including treatments essential for the management of numerous chronic diseases, such as cardiovascular disease and diabetes.

We found that decreases in chronic medication use were more pronounced in states with greater decreases in enrollment, which aligns with prior studies.^23,24^ Importantly, states that implemented policies designed to protect coverage for eligible beneficiaries successfully mitigated the reductions in medication use during the unwinding period. Thus, carefully designed Medicaid eligibility redetermination processes can successfully target those who are ineligible for Medicaid and prevent disruptions in chronic disease management for patients who remain eligible but who have difficulty navigating administrative procedures for eligibility redetermination. To minimize disruptions in care, states could leverage health care use data to target additional outreach and assistance navigating the redetermination process for patients receiving essential health care services. States that conduct interim data checks in between eligibility redeterminations might also use this data to exempt patients who might be more vulnerable if their care is disrupted. Such strategies will be particularly important in light of new laws that mandate Medicaid work requirements and increased the frequency of redeterminations for Medicaid eligibility.^9^

We found that the changes in Medicaid enrollment during and after the COVID-19 pandemic were larger than the changes in the number of prescriptions, aligning with a prior study.^25^ In addition, that the average number of prescriptions per enrollee decreased during the COVID-19 pandemic and increased during unwinding. One explanation for this pattern is that patients who maintained Medicaid as a result of the continuous coverage provision may have been healthier, had fewer chronic conditions, and used fewer medications than those who successfully navigated the frequent eligibility determinations before the COVID-19 pandemic (and would likely have remained enrolled even without the continuous coverage provision).^26^ Alternatively, some people who maintained Medicaid coverage during the COVID-19 pandemic may not have known they had Medicaid coverage.^4,27^ Better outreach and patient education may be necessary to ensure that continuous coverage policies—such as those now applying to postpartum individuals and children nationwide—produce commensurate gains in needed care.^28,29^

Because our dataset was limited to prescriptions paid for by Medicaid, we could not determine whether reductions in medication use occurred because patients were no longer taking those medicines or because payment for those medications switched to other sources (eg, private insurance, self-pay). One study^25^ found that the reduction in Medicaid-reimbursed prescriptions during unwinding was offset by an increasing number of prescriptions reimbursed by commercial insurance. However, even if patients who lost Medicaid found alternative prescription drug coverage, they would likely face much higher cost-sharing obligations and may have experienced gaps or formulary changes that disrupted medication access and adherence.^7,30^ For example, loss of medication cost-sharing subsidies for patients dual-enrolled in Medicaid and Medicare was associated with an increase in mortality.^31^

Chronic disease medication use did not decrease after the onset of the COVID-19 pandemic, a finding that has been observed in other studies and which differentiates medications from other health care services that faced important interruptions during the pandemic.^32^ However, the use of medications for acute conditions dropped substantially, driven largely by the number of antimicrobial prescriptions. Changes in antibiotic prescribing during the COVID-19 pandemic has also been previously reported,^33^ and is likely multifactorial due to delayed elective procedures (which can require antibiotic prophylaxis), reduced propensity for patients to seek in-person care for viral sinus or ear infections that often generate inappropriate antibiotic prescriptions and self-resolve,^34^ and reduced transmission of certain infections due to pandemic mitigation measures. Use of pediatric-specific medications also declined at the start of the COVID-19 pandemic, likely because most pediatric medications are for acute conditions. A study that was limited to medications for chronic diseases in children showed results that more closely match our chronic disease results.^23^ We also observed a large increase in the use of contraceptive medications; this pattern may be correlated with the observed decrease in US birth rates related to the early waves of the COVID-19 pandemic.^35^ The large decrease in hepatitis C antiviral use may suggest that the COVID-19 pandemic interrupted access because use of these medications had been increasing in Medicaid prior to 2020.^15^

### Limitations

We stratified drugs into different therapeutic areas based on a well-established international classification scheme, but some medications have multiple uses. Modeled trends for enrollment and mediation use during unwinding were based on 4 quarterly data points, fewer than is typically used for time series models. Even with these limited data, the changes in enrollment and medication use were significant. However, early unwinding trends may not predict future trends. Finally, temporal changes identified in interrupted time series models could be subject to confounding by other co-occurring events; specifically, changes in medication use in 2020 can be attributed both to the continuous enrollment provision and reduced health care access during the early phase of the COVID-19 pandemic.

## Conclusions

Changes in Medicaid enrollment during the COVID-19 pandemic continuous enrollment provision were associated with smaller yet significant changes in the use of prescription medications. The use of chronic disease medications increased during the pandemic but decreased during unwinding; limited patient access to medications for chronic conditions could translate to important effects on patient outcomes. States that disenrolled fewer individuals or implemented coverage protection policies were able to mitigate these changes.

## Notes

Supplement 1. eMethods eFigure 1. Nine outlier state-quarters excluded from interrupted time series analyses eTable 1. World Health Organization Anatomical Therapeutic Chemical classification codes used to identify drugs for selected conditions eTable 2. Quartiles of net state Medicaid enrollment change from March 2023 – April 2024 eTable 3. Number of policies to protect patient access adopted by states during the unwinding period eTable 4. Changes in Medicaid enrollment and prescription drug use during and after the Medicaid continuous enrollment provision, with a 1 quarter lag at the end of the provision eTable 5. Unweighted changes in Medicaid enrollment and prescription drug use during and after the Medicaid continuous enrollment provision eTable 6. Changes in Medicaid enrollment and prescription drug use during and after the Medicaid continuous enrollment provision, excluding 9 states that expanded Medicaid during the study period eTable 7. Changes in Medicaid enrollment and prescription drug use during the Medicaid continuous enrollment provision and unwinding, controlling for state unemployment rate and Medicaid expansion status eTable 8. Changes in number of prescriptions per Medicaid enrollee during the Medicaid continuous enrollment provision and unwinding eTable 9. Changes in Medicaid enrollment and medication use, stratified by state characteristics, excluding 9 states that expanded Medicaid between 2018Q1 and 2024Q1 eTable 10: Quartiles of net state Medicaid enrollment change from March 2023 – April 2024, excluding 9 states* that expanded Medicaid during the study period eFigure 2. Distribution of drugs used for pediatric patients eFigure 3. Changes in Medicaid Enrollment and Prescription Drug Use by Drug Class from 2018-2024 eFigure 4. Changes in Pediatric Medicaid Enrollment and Prescription Drug Use from 2018-2024

Supplement 2. Data Sharing Statement
