# Supplementary material for: Changes in Medication Use During Medicaid Continuous Enrollment and Unwinding
Source: JAMA Health Forum. 2026 Jan 2;7(1):e255890. doi: 10.1001/jamahealthforum.2025.5890 (PMC12761332; doi:10.1001/jamahealthforum.2025.5890)
Supplement: Supplement 1. — eMethods eFigure 1. Nine outlier state-quarters excluded from interrupted time series analyses eTable 1. World Health Organization Anatomical Therapeutic Chemical classification codes used to identify drugs for selected conditions eTable 2. Quartiles of net state Medicaid enrollment change from March 2023 – April 2024 eTable 3. Number of policies to protect patient access adopted by states during the unwinding period eTable 4. Changes in Medicaid enrollment and prescription drug use during and after the Medicaid continuous enrollment provision, with a 1 quarter lag at the end of the provision eTable 5. Unweighted changes in Medicaid enrollment and prescription drug use during and after the Medicaid continuous enrollment provision eTable 6. Changes in Medicaid enrollment and prescription drug use during and after the Medicaid continuous enrollment provision, excluding 9 states that expanded Medicaid during the study period eTable 7. Changes in Medicaid enrollment and prescription drug use during the Medicaid continuous enrollment provision and unwinding, controlling for state unemployment rate and Medicaid expansion status eTable 8. Changes in number of prescriptions per Medicaid enrollee during the Medicaid continuous enrollment provision and unwinding eTable 9. Changes in Medicaid enrollment and medication use, stratified by state characteristics, excluding 9 states that expanded Medicaid between 2018Q1 and 2024Q1 eTable 10: Quartiles of net state Medicaid enrollment change from March 2023 – April 2024, excluding 9 states* that expanded Medicaid during the study period eFigure 2. Distribution of drugs used for pediatric patients eFigure 3. Changes in Medicaid Enrollment and Prescription Drug Use by Drug Class from 2018-2024 eFigure 4. Changes in Pediatric Medicaid Enrollment and Prescription Drug Use from 2018-2024 [file jamahealthforum-e255890-s001.pdf]

Supplemental Online Content

Rome BN, Han J, McIntyre A, et al. Changes in medication use during Medicaid continuous enrollment and unwinding. *JAMA Health Forum*. 2025;7(1):e255890. doi:10.1001/jamahealthforum.2025.5890

eMethods
eFigure 1. Nine outlier state-quarters excluded from interrupted time series analyses
eTable 1. World Health Organization Anatomical Therapeutic Chemical classification codes used to identify drugs for selected conditions
eTable 2. Quartiles of net state Medicaid enrollment change from March 2023 – April 2024
eTable 3. Number of policies to protect patient access adopted by states during the unwinding period
eTable 4. Changes in Medicaid enrollment and prescription drug use during and after the Medicaid continuous enrollment provision, with a 1 quarter lag at the end of the provision
eTable 5. Unweighted changes in Medicaid enrollment and prescription drug use during and after the Medicaid continuous enrollment provision
eTable 6. Changes in Medicaid enrollment and prescription drug use during and after the Medicaid continuous enrollment provision, excluding 9 states that expanded Medicaid during the study period
eTable 7. Changes in Medicaid enrollment and prescription drug use during the Medicaid continuous enrollment provision and unwinding, controlling for state unemployment rate and Medicaid expansion status
eTable 8. Changes in number of prescriptions per Medicaid enrollee during the Medicaid continuous enrollment provision and unwinding
eTable 9. Changes in Medicaid enrollment and medication use, stratified by state characteristics, excluding 9 states that expanded Medicaid between 2018Q1 and 2024Q1
eTable 10: Quartiles of net state Medicaid enrollment change from March 2023 – April 2024, excluding 9 states* that expanded Medicaid during the study period
eFigure 2. Distribution of drugs used for pediatric patients
eFigure 3. Changes in Medicaid Enrollment and Prescription Drug Use by Drug Class from 2018-2024
eFigure 4. Changes in Pediatric Medicaid Enrollment and Prescription Drug Use from 2018-2024

This supplemental material has been provided by the authors to give readers additional information about their work.

© 2025 Rome BN et al. *JAMA Health Forum*.

© 2025 Rome BN et al. *JAMA Health Forum*.

eMethods

Enrollment data

We obtained monthly state Medicaid and CHIP enrollment data from public CMS files. Quarterly mean values were calculated to match the reporting of Medicaid drug use. Enrollment was obtained for the overall population (all ages) and for pediatric populations (ages 18 years or under). Two states (Tennessee, Arizona) lacked pediatric-specific enrollment for some of the study period, and these missing state-quarters were excluded from the pediatric-specific analyses.

Pediatric-specific medications

To understand whether trends during Medicaid unwinding were different for children, we identified a list of drugs with at least 50 reimbursed claims in the 2020 TAF file and for which at least 80% of claims were dispensed to patients aged 0-18 years old. We selected the 80% threshold by reviewing the overall distribution of drugs (**eFigure 1**); more than half of drugs with at least 80% pediatric use were oral liquid or chewable tablet formulations.

Number of prescriptions and outlier removal

Most state Medicaid programs limit prescriptions to 30 days' supply or less, but some of these restrictions were relaxed during the COVID-19 pandemic to allow prescriptions with 90 days' supply (Alpern JD, et al. *J Manag Care spec Pharm* 2021. 10.18553/jmcp.2021.27.10.1489). To prevent this change from artifactually lowering the number of reimbursed prescriptions over time, we measured changes in the number of units (e.g., tablets, milliliters) reimbursed, which are not sensitive to temporal changes in prescription days' supply. To allow comparison between drugs, we then converted the number of units to an estimated number of reimbursed prescriptions, based on the ratio of units per prescription in the first 4 quarters of available data (e.g., the 4 quarters of 2018 for drugs available throughout the entire study period).

The state-reported Medicaid drug use data contains errors that can result in outliers. We addressed this in two ways. First, we excluded quarters for specific drugs in a state if the ratio of units to prescriptions was more than 100 times higher than the median ratio for that drug in that state across all quarters in the study; this resulted in removing 543 (0.005%) out of 11.9 million drug-state-quarters of data. These outliers were removed at the drug level before results were aggregated and are reflect in all of the data presented in the figure.

Second, we removed 9 outlier state quarters data from the interrupted time series models. These were identified based on an interrupted time series model of total medication use; unlike the models used in the primary analysis, the model used to identify outliers was not weighted by state Medicaid enrollment, as we wanted to also excluded outliers in smaller states. A state-quarter data point was classified as an outlier and excluded from the analyses if it met either of the following criteria:

- Absolute standardized residual > 3, or
- Leverage > 0.20 (this threshold was determined as 2(p/n), where p represents the number of predictors (5 covariates) and n represents the number of observations (51 states).

Nine quarters of data from 6 states met these criteria (eFigure 1), and these quarters were excluded from all subsequent interrupted time series models.

Interrupted Time Series Model

The models used to measure \changes in Medicaid enrollment and prescription use were as follows:

$$\ln(Y_{ts}) = \beta_0 + \beta_1 \times t + \beta_2 \times t_{cc} + \beta_3 \times i_{cc} + \beta_4 \times t_u + s_i + \epsilon_t$$

Y_{ts} = the number of enrollees or number of prescriptions reimbursed during time (quarter) t in state s.

t = calendar quarter starting from 2018Q1, where 1 = 2018Q1, 2 = 2018Q2, etc.

t_{cc} = calendar quarter after the continuous enrollment provision, where all quarters through 2020Q1 = 0, 2020Q2 = 1, 2020Q3 = 2, etc.

i_{cc} = indicator variable for time before vs. after the continuous enrollment provision, where all quarters through 2020Q1 = 0 and all quarters from 2020Q2 and onward = 1.

t_u = calendar quarter after the unwinding (post-continuous enrollment) period, where all quarters through 2023Q1 = 0, 2023Q2 = 1, 2023Q3 = 2, etc.

s_i = state fixed effects

© 2025 Rome BN et al. *JAMA Health Forum*.

eFigure 1: Nine outlier state-quarters excluded from interrupted time series analyses

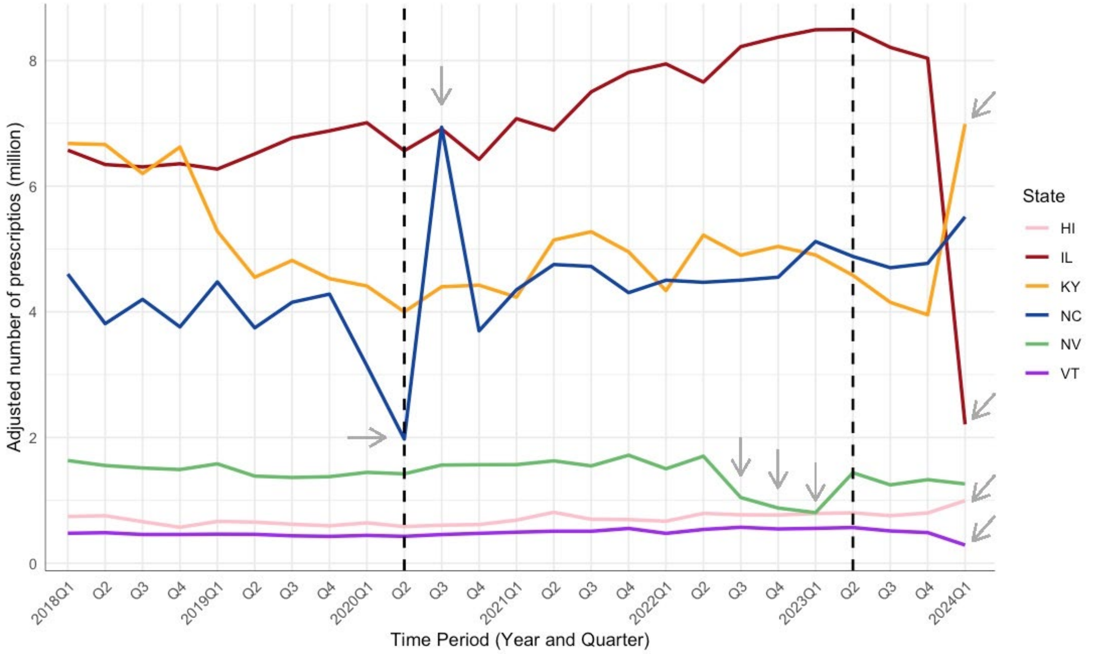

The figure shows trends in the total number of adjusted prescriptions reimbursed in six states that contained outliers (eMethods). The 9 outlier quarters are denoted by arrows and were excluded from all subsequent interrupted time series models.

© 2025 Rome BN et al. *JAMA Health Forum*.

eTable 1: World Health Organization Anatomical Therapeutic Chemical classification codes used to identify drugs for selected conditions

**Chronic conditions**	**WHO-ATC Codes**
Diabetes	A10* (Drugs used in diabetes)
Cardiovascular disease	B01* (Antithrombotic agents)<br>C01* (Cardiac therapy)<br>C03* (Diuretics)<br>C07* (Beta blocking agents)<br>C08* (Calcium channel blockers)<br>C09* (Renin-angiotensin system agents)<br>C10* (Lipid modifying agents)
Cancer	L01* (Antineoplastic agents)
Psychiatric disease	N05A* (Antipsychotics)<br>N06A* (Antidepressants)<br>N06B* (Psychostimulants)
HIV	J05AE* (Protease inhibitors)<br>J05AF* (NRTIs)<br>J05AG* (NNRTIs)<br>J05AJ* (Integrase inhibitors)<br>J05AE01-10 (Protease inhibitors)<br>J05AR* (Combination HIV antivirals)<br>J05AX07 (enfuviride)<br>J05AX09 (maraviroc)<br>J05AX23 (Ibalizumab)<br>J05AX29 (Fostemsavir)<br>J05AX31 (Lencapavir)
**Acute conditions**	
Anti-infectives	J01* (Antibacterials)<br>J02* (Antimycotics)<br>J04* (Antimycobacterials)<br>J05* (Antvirals)<br>P* (Parasitics)<br>S01A* (Ophthalmalogicsals, antiinfectives)<br>S01C* (Ophthalmalogicsals, anti-inflammatory and antiinfectives)<br>S02AA* (Otologicals, antiinfectives)<br>S02CA* (Otologicals, corticosteroid and antiinfectives in combination)
Cold and allergy medications	R01A* (Decongestants and other nasal preparations for topical use)<br>R01B* (Nasal decongestants for systemic use)<br>R05* (Cough and cold preparations)<br>R06* (Antihistamines for systemic use)<br>S01G* (Decongestants and antiallergics)
**Other conditions of interest**	
Asthma & chronic obstructive pulmonary disease	R03* (Drugs for obstructive airway diseases)
Contraceptives	G03A* (Hormonal contraceptives for systemic use)
Hepatitis C antivirals	J05AP* (Hepatitis C Antivirals)
Covid-19 antivirals	J05AB16 (Remdesivir)<br>J05AB18 (Molnupiravir)<br>J05AE30 (Nirmatrelvir/ritonavir)

eTable 2: Quartiles of net state Medicaid enrollment change from March 2023 – April 2024

**Quartile**	**Net enrollment change**	**States**
1st	>19.5% decrease	AR, CO, FL, IA, ID, MT, ND, NH, OK, TX, UT, WV, WY
2nd	13.9%, 19.4% decrease	AL, GA, KS, LA, MA, MI, MN, MS, OH, PA, TN, VT, WA
3rd	9.5%, 13.8% decrease	AZ, DC, DE, IL, IN, MO, NE, NJ, NM, RI, SC, SD, WI
4th	≤9.4% decrease	AK, CA, CT, HI, KY, MD, ME, NC, NV, NY, OR, VA

© 2025 Rome BN et al. *JAMA Health Forum*.

eTable 3: Number of policies to protect patient access adopted by states during the unwinding period

# Policies [a]	States
0-1	AL, FL, OH, OK, SC, TX
2	AR, DC, KS, MA, MD, MD, MS, MT, NC, NC, NH, NM, NV, SD, TN, UT, VA, VT, WI, WY
3	AZ, CO, CT, GA, HI, IA, ID, MI, MN, MO, ND, NJ, NY, RI, WA, OR
4	AK, CA, DE, IL, IN, KY, LA, ME, NE, PA, WV

[a] Policies included Medicaid expansion, spreading out redeterminations over a full year, planning to take a full year to complete the unwinding process, and making use of more sources of income data to reduce beneficiaries' reporting burden. **Source:** McIntyre A, Aboulafia G, Sommers BD. Preliminary Data on "Unwinding" Continuous Medicaid Coverage. *N Engl J Med*. 2023;389(24):2215-2217. doi:10.1056/NEJMp2311336

© 2025 Rome BN et al. *JAMA Health Forum*.

eTable 4. Changes in Medicaid enrollment and prescription drug use during and after the Medicaid continuous enrollment provision, with a 1 quarter lag at the end of the provision

Outcome	Baseline trend, before the continuous enrollment provision [a], % per quarter (95% CI)	Level change, start of continuous enrollment provision [a], % per quarter (95% CI)	Trend change, during the continuous enrollment provision [a], % per quarter (95% CI)	Trend change, during unwinding [a], % per quarter (95% CI)
**Enrollment**	-0.33 (-0.54 to -0.12)**	4.12 (3.00 to 5.26)***	2.38 (2.11 to 2.65)***	-4.84 (-6.03 to -3.64)***
**Medication Use**	-0.11 (-0.55 to 0.34)	-5.47 (-8.33 to -2.52)***†	1.87 (1.26 to 2.49)***†	-3.97 (-5.68 to -2.24)***†
**Chronic diseases**	0.26 (-0.18 to 0.71)†	4.88 (2.54 to 7.28)***	1.11 (0.66 to 1.56)***†	-3.80 (-5.25 to -2.32)***†
Cancer	0.50 (-0.95 to 1.97)	0.98 (-5.21 to 7.57)	-1.13 (-2.52 to 0.28)†	-3.17 (-5.92 to -0.33)*
Cardiovascular	0.22 (-0.32 to 0.76)†	6.92 (4.03 to 9.88)***†	0.90 (0.35 to 1.44)**†	-4.17 (-5.50 to -2.82)***
Diabetes	0.50 (0.03 to 0.98)*†	6.07 (3.32 to 8.89)***	1.55 (1.10 to 2.00)***†	-3.89 (-5.80 to -1.95)***†
HIV	-2.06 (-2.90 to -1.22)***†	-0.85 (-3.38 to 1.76)†	3.68 (2.56 to 4.82)***†	-3.43 (-5.09 to -1.73)***†
Psychiatric [b]	0.37 (-0.00 to 0.74)	2.99 (0.84 to 5.19)**	1.10 (0.69 to 1.51)***	-3.45 (-4.94 to -1.94)***
**Acute conditions**	-0.35 (-0.75 to 0.04)	-28.62 (-31.66 to -25.45)***†	4.05 (3.14 to 4.97)***†	-5.77 (-8.37 to -3.09)***†
Anti-infectives	-0.41 (-0.78 to -0.04)*	-36.94 (-40.17 to -33.53)***†	5.50 (4.52 to 6.49)***†	-7.32 (-9.88 to -4.69)***†
Cold and Allergy	-0.19 (-0.70 to 0.33)	-15.53 (-20.16 to -10.64)***†	1.98 (1.07 to 2.91)***	-3.74 (-6.38 to -1.03)**†
**Other drugs**				
Asthma and COPD	0.30 (-0.13 to 0.73)†	-10.79 (-15.16 to -6.20)***†	1.15 (0.73 to 1.57)***†	-3.46 (-4.88 to -2.02)***†
HCV antivirals	0.77 (-2.52 to 4.17)	-23.30 (-28.24 to -18.03)***†	-0.34 (-4.24 to 3.71)†	-2.83 (-7.97 to 2.60)
Contraceptives	-0.25 (-0.80 to 0.30)	14.31 (7.97 to 21.03)***†	1.03 (0.26 to 1.79)**†	-6.44 (-9.01 to -3.79)***†
Other [c]	-0.37 (-0.90 to 0.16)	-3.22 (-6.21 to -0.13)*	1.81 (1.13 to 2.48)***	-3.36 (-4.89 to -1.82)***
**Pediatric**				
Enrollment	-0.42 (-0.60 to -0.24)***	2.82 (1.81 to 3.85)***	1.63 (1.38 to 1.87)***	-3.41 (-4.53 to -2.28)***
Medication Use	0.19 (-0.14 to 0.52)†	-43.84 (-48.31 to -38.99)***†	4.20 (3.54 to 4.86)***†	-7.51 (-9.61 to -5.37)***†

Abbreviations: COPD = chronic obstructive pulmonary disease, HCV= hepatitis C, HIV = Human immunodeficiency virus
* p<0.05, ** p<0.01, *** p<0.001
† p<0.05 for the post-estimation coefficient test, compared to the coefficient for change in enrollment.
[a] The baseline period was 2018Q1-2020Q1, the continuous enrollment period was 2020Q2-2023Q1, and the unwinding period was 2023Q3-2024Q1. Data from 2023Q2 was excluded in this analysis.
[b] Includes antidepressants, antipsychotics, and stimulants
[c] Includes all medications not in one of the specified categories

eTable 5. Unweighted changes in Medicaid enrollment and prescription drug use during and after the Medicaid continuous enrollment provision

Outcome	Baseline trend, before the continuous enrollment provision [a], % per quarter (95% CI)	Level change, start of continuous enrollment provision [a], % per quarter (95% CI)	Trend change, during the continuous enrollment provision [a], % per quarter (95% CI)	Trend change, during unwinding [a], % per quarter (95% CI)
**Enrollment**	-0.18 (-0.45 to 0.09)	4.74 (3.67 to 5.82)***	2.44 (2.08 to 2.81)***	-5.68 (-6.44 to -4.91)***
**Medication Use**	0.14 (-0.50 to 0.78)	-3.16 (-5.57 to -0.69)*†	1.87 (1.12 to 2.63)***†	-5.40 (-6.37 to -4.43)***
**Chronic diseases**	0.58 (-0.10 to 1.27)†	6.40 (3.85 to 9.02)***	1.25 (0.50 to 2.00)**†	-5.34 (-6.32 to -4.36)***
Cancer	0.70 (-1.02 to 2.46)	5.31 (-2.95 to 14.27)	-2.00 (-3.86 to -0.11)*†	-4.52 (-8.58 to -0.28)*
Cardiovascular	0.65 (-0.20 to 1.50)†	9.08 (5.87 to 12.37)***†	0.96 (0.02 to 1.91)*†	-5.81 (-6.99 to -4.62)***
Diabetes	0.94 (0.20 to 1.69)*†	7.02 (4.08 to 10.04)***	1.61 (0.77 to 2.45)***†	-5.92 (-7.40 to -4.41)***
HIV	-1.26 (-2.46 to -0.05)*†	2.64 (-1.97 to 7.47)	3.55 (1.90 to 5.22)***†	-4.56 (-6.13 to -2.97)***
Psychiatric [b]	0.51 (-0.09 to 1.11)	4.54 (2.11 to 7.02)***	1.32 (0.67 to 1.97)***	-4.81 (-5.68 to -3.93)***
**Acute conditions**	-0.28 (-0.81 to 0.26)	-27.43 (-29.55 to -25.24)***†	4.16 (3.41 to 4.92)***†	-7.43 (-8.64 to -6.20)***†
Anti-infectives	-0.24 (-0.79 to 0.31)	-36.52 (-38.72 to -34.25)***†	5.55 (4.71 to 6.40)***†	-8.79 (-10.10 to -7.46)***†
Cold and Allergy	-0.27 (-0.83 to 0.29)	-11.48 (-14.63 to -8.22)***†	2.04 (1.29 to 2.78)***	-5.43 (-6.63 to -4.21)***
**Other drugs**				
Asthma and COPD	0.37 (-0.15 to 0.90)†	-6.60 (-9.69 to -3.40)***†	0.95 (0.35 to 1.55)**†	-4.90 (-6.14 to -3.64)***
HCV antivirals	2.94 (0.07 to 5.89)*†	-21.47 (-28.44 to -13.83)***†	-1.78 (-4.96 to 1.51)†	-3.41 (-7.39 to 0.74)
Contraceptives	0.23 (-0.55 to 1.01)	11.97 (7.44 to 16.70)***†	1.15 (0.36 to 1.96)**†	-7.16 (-8.82 to -5.46)***†
Other [c]	-0.12 (-0.82 to 0.58)	-0.29 (-2.71 to 2.20)	1.71 (0.87 to 2.55)***	-4.73 (-5.75 to -3.71)***
**Pediatric**				
Enrollment	-0.37 (-0.54 to -0.19)***	3.32 (2.53 to 4.12)***	1.71 (1.46 to 1.97)***	-4.07 (-4.77 to -3.36)***
Medication Use	0.07 (-0.31 to 0.46)	-38.41 (-40.56 to -36.17)***†	3.73 (3.08 to 4.40)***†	-8.38 (-9.60 to -7.15)***†

Abbreviations: COPD = chronic obstructive pulmonary disease, HCV= hepatitis C, HIV = Human immunodeficiency virus

* p<0.05, ** p<0.01, *** p<0.001

† p<0.05 for the post-estimation coefficient test, compared to the coefficient for the change in enrollment

[a] The baseline period was 2018Q1-2020Q1, the continuous enrollment period was 2020Q2-2023Q1, and the unwinding period was 2023Q2-2024Q1.

[b] Includes antidepressants, antipsychotics, and stimulants

[c] Includes all medications not in one of the specified categories

© 2025 Rome BN et al. *JAMA Health Forum*.

eTable 6. Changes in Medicaid enrollment and prescription drug use during and after the Medicaid continuous enrollment provision, excluding 9 states that expanded Medicaid during the study period. [a]

Outcome	**Baseline trend, before the continuous enrollment provision** [b], % per quarter (95% CI)	**Level change, start of continuous enrollment provision** [b], % per quarter (95% CI)	**Trend change, during the continuous enrollment provision** [b], % per quarter (95% CI)	**Trend change, during unwinding** [b], % per quarter (95% CI)
**Enrollment**	-0.41 (-0.55 to -0.27)***	3.82 (2.68 to 4.98)***	2.41 (2.20 to 2.63)***	-4.90 (-6.16 to -3.63)***
**Medication Use**	-0.20 (-0.57 to 0.17)	-5.68 (-8.45 to -2.83)***†	1.85 (1.25 to 2.46)***†	-3.91 (-5.83 to -1.95)***†
**Chronic diseases**	0.16 (-0.20 to 0.53)†	4.65 (2.55 to 6.80)***	1.07 (0.68 to 1.46)***†	-3.80 (-5.49 to -2.08)***†
Cancer	0.46 (-1.07 to 2.02)	0.64 (-5.88 to 7.61)	-1.20 (-2.63 to 0.26)†	-2.60 (-5.50 to 0.39)†
Cardiovascular	0.06 (-0.37 to 0.49)†	6.59 (3.93 to 9.31)***†	0.89 (0.43 to 1.34)***†	-4.12 (-5.62 to -2.60)***†
Diabetes	0.37 (0.01 to 0.73)*†	5.65 (3.23 to 8.14)***	1.56 (1.21 to 1.92)***†	-3.94 (-6.17 to -1.66)***†
HIV	-2.29 (-3.00 to -1.57)***†	0.01 (-2.15 to 2.22)†	3.43 (2.42 to 4.45)***†	-3.11 (-4.88 to -1.30)***†
Psychiatric [c]	0.33 (-0.00 to 0.66)	2.86 (0.92 to 4.84)**	1.01 (0.59 to 1.42)***	-3.42 (-5.18 to -1.62)***
**Acute conditions**	-0.40 (-0.75 to -0.06)*	-28.73 (-31.84 to -25.49)***†	4.01 (3.07 to 4.96)***†	-5.64 (-8.45 to -2.75)***
Anti-infectives	-0.50 (-0.79 to -0.21)***	-36.56 (-40.01 to -32.91)***†	5.35 (4.33 to 6.37)***†	-6.98 (-9.75 to -4.13)***†
Cold and Allergy	-0.18 (-0.68 to 0.33)	-16.67 (-21.15 to -11.93)***†	2.11 (1.13 to 3.09)***	-3.94 (-6.80 to -1.00)**†
**Other drugs**				
Asthma and COPD	0.31 (-0.10 to 0.72)†	-10.88 (-15.15 to -6.40)***†	0.98 (0.60 to 1.37)***†	-3.18 (-4.75 to -1.58)***†
HCV	0.63 (-2.79 to 4.17)	-24.77 (-29.72 to -19.48)***†	-0.23 (-4.29 to 4.00)†	-3.07 (-8.51 to 2.69)
Contraceptives	-0.37 (-0.89 to 0.15)	14.69 (8.17 to 21.60)***†	0.95 (0.11 to 1.79)*†	-6.30 (-9.22 to -3.29)***†
Other [d]	-0.50 (-0.96 to -0.03)*	-3.55 (-6.31 to -0.71)*	1.84 (1.18 to 2.50)***	-3.30 (-5.02 to -1.55)***
**Pediatric**				
Enrollment	-0.43 (-0.60 to -0.25)***	2.44 (1.48 to 3.41)***	1.63 (1.38 to 1.88)***	-3.40 (-4.61 to -2.17)***
Medication Use	0.20 (-0.13 to 0.54)†	-44.13 (-48.56 to -39.32)***†	4.05 (3.39 to 4.73)***†	-7.14 (-9.40 to -4.82)***†

Abbreviations: COPD = chronic obstructive pulmonary disease, HCV= hepatitis C, HIV = Human immunodeficiency virus

* p<0.05, ** p<0.01, *** p<0.001

† p<0.05 for the post-estimation coefficient test, compared to the coefficient for the change in enrollment

[a] The nine states that expanded Medicaid during the study period were NE, OK, MO, SD, NC, VA, ME, ID, and UT.

[b] The baseline period was 2018Q1-2020Q1, the continuous enrollment period was 2020Q2-2023Q1, and the unwinding period was 2023Q2-2024Q1.

[c] Includes antidepressants, antipsychotics, and stimulants

[d] Includes all medications not in one of the specified categories

© 2025 Rome BN et al. *JAMA Health Forum*.

eTable 7. Changes in Medicaid enrollment and prescription drug use during the Medicaid continuous enrollment provision and unwinding, controlling for state unemployment rate and Medicaid expansion status

Outcome	Baseline trend, before the continuous enrollment provision [a], % per quarter (95% CI)	Level change, start of continuous enrollment provision [a], % per quarter (95% CI)	Trend change, during the continuous enrollment provision [a], % per quarter (95% CI)	Trend change, during unwinding [a], % per quarter (95% CI)
**Enrollment**	-0.40 (-0.54 to -0.26)***	7.11 (5.74 to 8.50)***	2.15 (1.85 to 2.46)***	-4.88 (-5.93 to -3.81)***
**Estimated number of prescriptions**	-0.19 (-0.58 to 0.21)	-4.04 (-8.75 to 0.91)†	1.76 (1.18 to 2.34)***†	-4.35 (-5.84 to -2.84)***
**Chronic diseases**	0.17 (-0.21 to 0.55)†	6.80 (2.75 to 11.02)***	0.98 (0.56 to 1.40)***†	-4.23 (-5.53 to -2.91)***
Cancer	0.42 (-1.03 to 1.90)	0.18 (-11.19 to 13.01)	-1.03 (-2.70 to 0.67)†	-4.00 (-6.75 to -1.17)**
Cardiovascular	0.11 (-0.33 to 0.54)†	10.19 (5.52 to 15.07)***	0.66 (0.15 to 1.17)*†	-4.52 (-5.94 to -3.08)***
Diabetes	0.39 (0.01 to 0.77)*†	7.42 (2.45 to 12.63)**	1.51 (1.07 to 1.95)***†	-4.64 (-6.15 to -3.10)***
HIV	-2.22 (-2.89 to -1.56)***†	-6.76 (-12.74 to -0.37)*†	4.36 (2.91 to 5.82)***†	-6.08 (-8.32 to -3.79)***
Psychiatric [b]	0.29 (-0.06 to 0.65)	3.94 (0.19 to 7.84)*	1.04 (0.62 to 1.46)***	-3.90 (-5.06 to -2.74)***
**Acute conditions**	-0.41 (-0.77 to -0.05)*	-29.71 (-34.53 to -24.54)***†	4.23 (3.49 to 4.98)***†	-6.57 (-8.39 to -4.72)***†
Anti-infectives	-0.49 (-0.79 to -0.18)**	-37.20 (-41.60 to -32.47)***†	5.53 (4.62 to 6.45)***†	-7.96 (-9.89 to -6.00)***†
Cold and Allergy	-0.23 (-0.73 to 0.28)	-16.05 (-23.16 to -8.29)***†	2.13 (1.36 to 2.90)***	-4.31 (-6.31 to -2.27)***
**Other drugs**				
Asthma and COPD	0.25 (-0.17 to 0.67)†	-11.61 (-17.96 to -4.76)**†	1.21 (0.69 to 1.74)***†	-3.99 (-5.53 to -2.43)***†
HCV antivirals	0.54 (-2.64 to 3.81)	-13.54 (-22.82 to -3.15)*†	-1.29 (-5.56 to 3.17)†	-2.40 (-7.60 to 3.10)
Contraceptives	-0.37 (-0.86 to 0.13)	28.41 (19.59 to 37.87)***†	-0.12 (-1.20 to 0.98)†	-4.88 (-7.87 to -1.79)**
Other [c]	-0.47 (-0.96 to 0.03)	0.06 (-5.05 to 5.45)	1.51 (0.86 to 2.17)***	-3.43 (-4.90 to -1.94)***
**Pediatric**				
Enrollment	-0.45 (-0.62 to -0.28)***	5.43 (4.53 to 6.34)***	1.40 (1.10 to 1.71)***	-3.13 (-4.31 to -1.93)***
Medication Use	0.12 (-0.20 to 0.44)†	-39.10 (-44.41 to -33.28)***†	3.29 (2.46 to 4.14)***†	-6.23 (-8.35 to -4.05)***†

Abbreviations: COPD = chronic obstructive pulmonary disease, HCV= hepatitis C, HIV = Human immunodeficiency virus

* p<0.05, ** p<0.01, *** p<0.001

† p<0.05 for the post-estimation coefficient test, compared to the coefficient for the change in enrollment

[a] The baseline period was 2018Q1-2020Q1, the continuous enrollment period was 2020Q2-2023Q1, and the unwinding period was 2023Q2-2024Q1.

[b] Includes antidepressants, antipsychotics, and stimulants

[c] Includes all medications not in one of the specified categories

© 2025 Rome BN et al. *JAMA Health Forum*.

eTable 8. Changes in number of prescriptions per Medicaid enrollee during the Medicaid continuous enrollment provision and unwinding

Outcome	Baseline trend, before the continuous enrollment provision [a], % per quarter (95% CI)	Level change, start of continuous enrollment provision [a], % per quarter (95% CI)	Trend change, during the continuous enrollment provision [a], % per quarter (95% CI)	Trend change, during unwinding [a], % per quarter (95% CI)
**Estimated number of prescriptions per enrollee**	0.15 (-0.14 to 0.45)	-6.28 (-7.74 to -4.80)***	-0.39 (-0.76 to -0.01)*	0.73 (0.20 to 1.26)**
**Chronic diseases**	0.26 (0.09 to 0.44)**	0.38 (-0.24 to 1.01)	-0.55 (-0.74 to -0.36)***	0.49 (0.19 to 0.79)**
Cancer	0.00 (-0.00 to 0.01)	-0.01 (-0.05 to 0.03)	-0.02 (-0.03 to -0.01)***	0.02 (-0.00 to 0.04)
Cardiovascular	0.13 (0.00 to 0.26)*	0.65 (0.17 to 1.13)**	-0.37 (-0.52 to -0.22)***	0.19 (0.02 to 0.37)*
Diabetes	0.08 (0.04 to 0.12)***	0.14 (-0.03 to 0.30)	-0.07 (-0.11 to -0.02)**	0.09 (-0.06 to 0.24)
HIV	-0.02 (-0.03 to -0.01)***	-0.03 (-0.05 to -0.02)***	0.01 (0.00 to 0.01)***	0.02 (0.00 to 0.03)*
Psychiatric [b]	0.17 (0.09 to 0.25)***	-0.20 (-0.51 to 0.11)	-0.30 (-0.39 to -0.21)***	0.35 (0.22 to 0.47)***
**Acute conditions**	-0.03 (-0.15 to 0.10)	-9.25 (-10.52 to -7.97)***	0.41 (0.19 to 0.64)***	-0.20 (-0.58 to 0.19)
Anti-infectives	-0.03 (-0.09 to 0.03)	-7.84 (-8.93 to -6.75)***	0.47 (0.32 to 0.62)***	-0.34 (-0.59 to -0.09)**
Cold and Allergy	-0.00 (-0.10 to 0.09)	-2.89 (-3.71 to -2.06)***	-0.00 (-0.14 to 0.14)	0.12 (-0.08 to 0.33)
**Other drugs**				
Asthma and COPD	0.08 (0.02 to 0.14)*	-1.88 (-2.27 to -1.48)***	-0.17 (-0.23 to -0.12)***	0.22 (0.12 to 0.31)***
HCV antivirals	0.00 (-0.00 to 0.00)	-0.01 (-0.02 to -0.01)***	-0.00 (-0.00 to 0.00)	0.00 (-0.00 to 0.00)
Contraceptives	0.00 (-0.01 to 0.02)	0.34 (0.18 to 0.50)***	-0.06 (-0.08 to -0.03)***	-0.03 (-0.09 to 0.02)
Other [c]	-0.03 (-0.29 to 0.24)	-3.44 (-4.47 to -2.39)***	-0.30 (-0.61 to 0.01)	0.82 (0.48 to 1.15)***
**Pediatric prescriptions per CHIP enrollee**	0.16 (0.04 to 0.28)**	-13.50 (-14.90 to -12.07)***	0.50 (0.29 to 0.70)***	-0.88 (-1.29 to -0.46)***

© 2025 Rome BN et al. *JAMA Health Forum*.

eTable 9. Changes in Medicaid enrollment and medication use, stratified by state characteristics, excluding 9 states that expanded Medicaid between 2018Q1 and 2024Q1

Outcome	**Baseline trend, before the continuous enrollment provision,** % per quarter (95% CI)	**Level change, start of continuous enrollment provision,** % per quarter (95% CI)	**Trend change, during the continuous enrollment provision,** % per quarter (95% CI)	**Trend change, during unwinding,** % per quarter (95% CI)
**Quartiles by net state enrollment change from March 2023 to April 2024 [a]**				
**Enrollment**				
1st quartile	-0.56 (-0.77 to -0.34)***	4.23 (3.47 to 4.98)***	3.02 (2.60 to 3.43)***	-8.59 (-9.90 to -7.25)***
2nd quartile	-0.32 (-0.54 to -0.09)**	3.48 (2.28 to 4.70)***	2.37 (2.07 to 2.67)***†	-5.12 (-5.47 to -4.78)***†
3rd quartile	-0.23 (-0.56 to 0.10)	4.93 (3.53 to 6.36)***	2.24 (1.89 to 2.59)***†	-4.93 (-5.33 to -4.53)***†
4th quartile	-0.46 (-0.67 to -0.26)***	3.20 (1.07 to 5.37)**	2.22 (1.98 to 2.46)***†	-2.83 (-3.22 to -2.44)***†
**Number of prescriptions for chronic disease medications**				
1st quartile	0.29 (-0.43 to 1.01)	4.19 (-0.38 to 8.96)	1.09 (0.61 to 1.58)***	-6.60 (-7.52 to s-5.66)***
2nd quartile	0.59 (0.19 to 0.99)**	6.13 (4.17 to 8.13)***	0.88 (0.26 to 1.50)**	-4.53 (-5.42 to -3.63)***†
3rd quartile	0.20 (-0.42 to 0.82)	6.02 (1.80 to 10.41)**	1.32 (0.75 to 1.90)***	-4.33 (-4.90 to -3.76)***†
4th quartile	-0.10 (-0.85 to 0.65)	3.62 (0.43 to 6.92)*	1.00 (0.20 to 1.81)*	-1.56 (-3.54 to 0.46)†
**Number of coverage-promoting policies**				
**Enrollment**				
0-1	-0.57 (-0.81 to -0.33)***	4.25 (2.82 to 5.70)***	2.94 (2.45 to 3.44)***	-7.53 (-9.82 to -5.18)***
2	-0.35 (-0.58 to -0.12)**	3.59 (2.05 to 5.15)***	2.36 (2.07 to 2.65)***†	-5.34 (-6.35 to -4.32)***†
3	-0.27 (-0.45 to -0.09)**	4.12 (3.51 to 4.73)***	2.30 (1.99 to 2.62)***†	-4.74 (-5.97 to -3.49)***†
4	-0.47 (-0.71 to -0.23)***	3.42 (0.72 to 6.19)*	2.25 (2.01 to 2.49)***†	-3.38 (-4.45 to -2.30)***†
**Number of prescriptions for chronic disease medications**				
0-1	0.00 (-0.33 to 0.34)	3.63 (-1.03 to 8.51)	1.19 (1.01 to 1.37)***	-5.94 (-7.50 to -4.35)***
2	0.18 (-0.73 to 1.10)	8.09 (2.07 to 14.46)**	1.30 (0.39 to 2.21)**	-5.35 (-7.11 to -3.55)***
3	0.26 (-0.27 to 0.79)	5.30 (3.19 to 7.46)***	1.07 (0.60 to 1.54)***	-3.34 (-4.76 to -1.90)***†
4	0.16 (-0.68 to 1.01)	3.23 (-0.03 to 6.60)	0.91 (0.07 to 1.77)*	-2.26 (-5.08 to 0.66)†

* p<0.05, ** p<0.01, *** p<0.001,
† p<0.05 for the post-estimation coefficient test, compared to the coefficient for the change in the 1st quartile or 0-1 policy group.
[a] 1st quartile includes the quartile of states with the largest negative net changes (e.g., largest decrease) in net Medicaid enrollment from March 2023 to April 2024. 4th quartile includes states with the smallest decreases (or increases) in enrollment

© 2025 Rome BN et al. *JAMA Health Forum*.

eTable 10: Quartiles of net state Medicaid enrollment change from March 2023 – April 2024, excluding 9 states* that expanded Medicaid during the study period

Quartile	Net enrollment change	States
1st	>18.5% decrease	AR, CO, FL, GA, IA, MT, ND, NH, TX, WV, WY
2nd	14.2%, 17.4% decrease	AL, KS, LA, MA, MI, MN, MS, TN, VT, WA
3rd	9.8%, 14.1% decrease	AZ, DE, IN, NJ, NM, OH, PA, RI, SC, WI
4th	≤9.7% decrease	AK, CA, CT, DC, HI, IL, KY, MD, NV, NY, OR

* The 9 states that expanded Medicaid included NE, OK, MO, SD, NC, VA, ME, UT, ID.

© 2025 Rome BN et al. *JAMA Health Forum*.

© 2025 Rome BN et al. *JAMA Health Forum*.

eFigure 2. Distribution of drugs used for pediatric patients

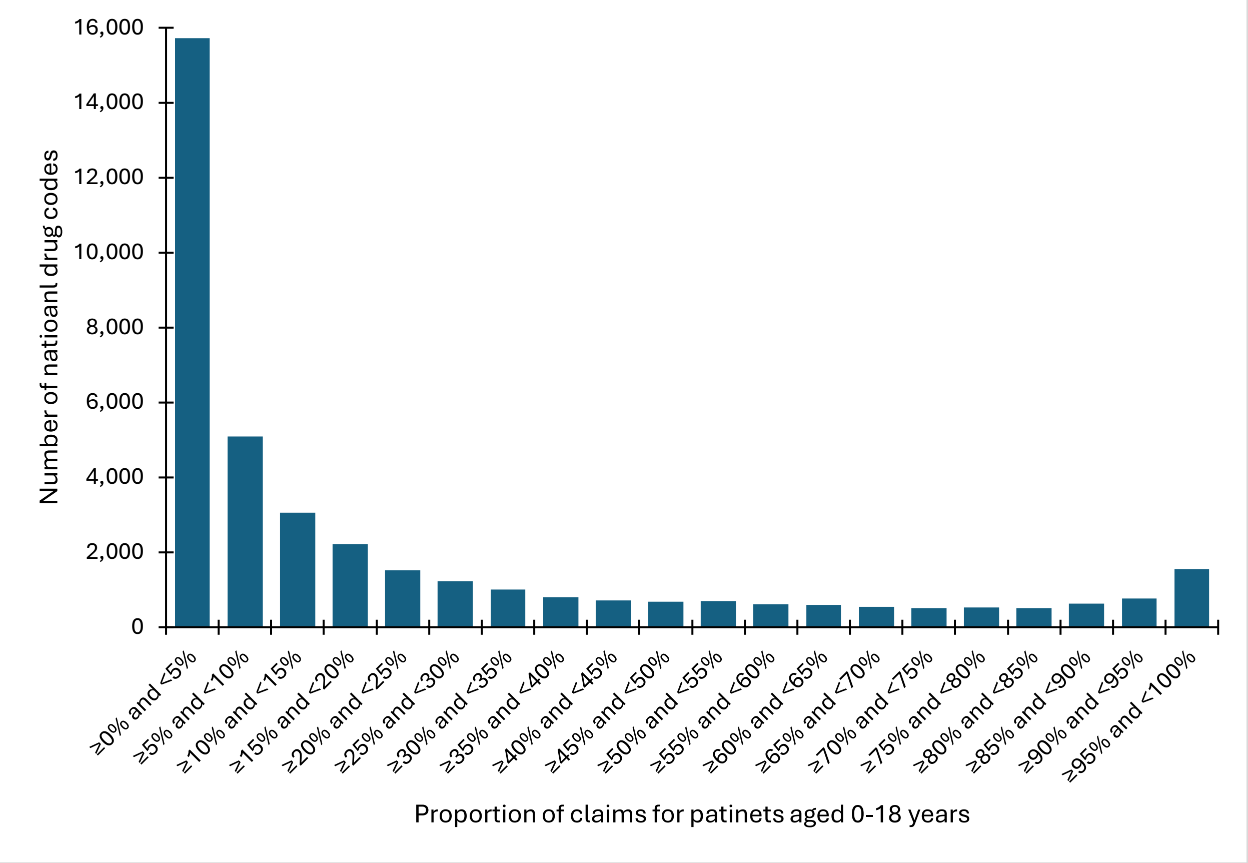

eFigure 3. Changes in Medicaid Enrollment and Prescription Drug Use by Drug Class from 2018-2024

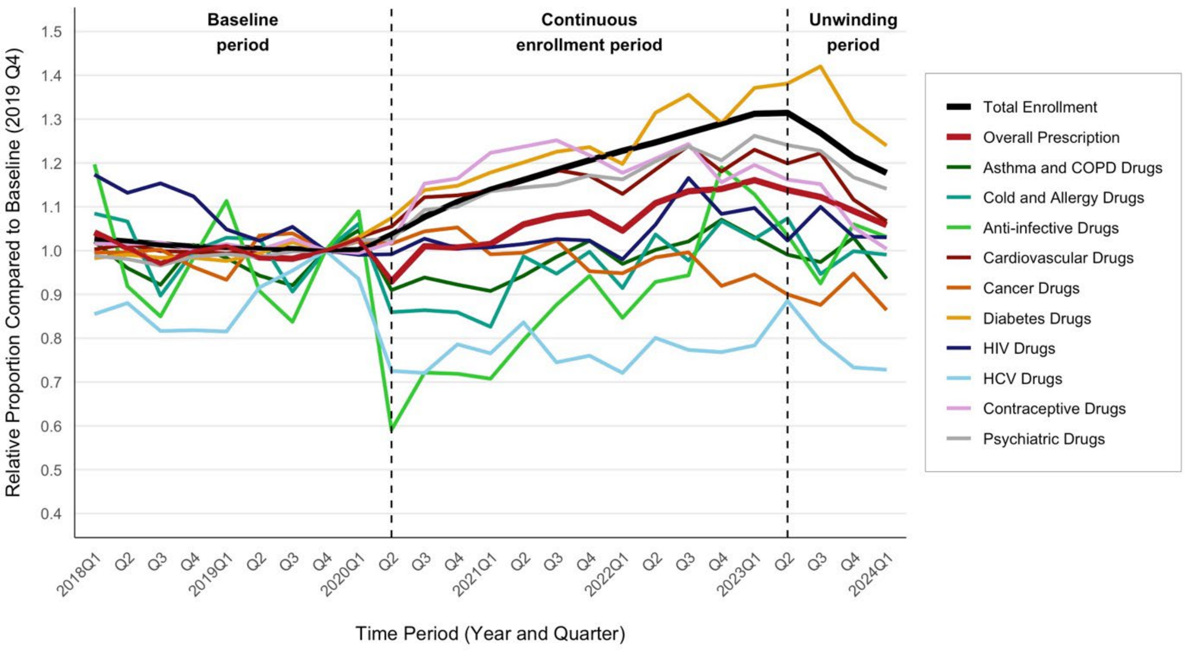

© 2025 Rome BN et al. *JAMA Health Forum*.

eFigure 4. Changes in Pediatric Medicaid Enrollment and Prescription Drug Use from 2018-2024

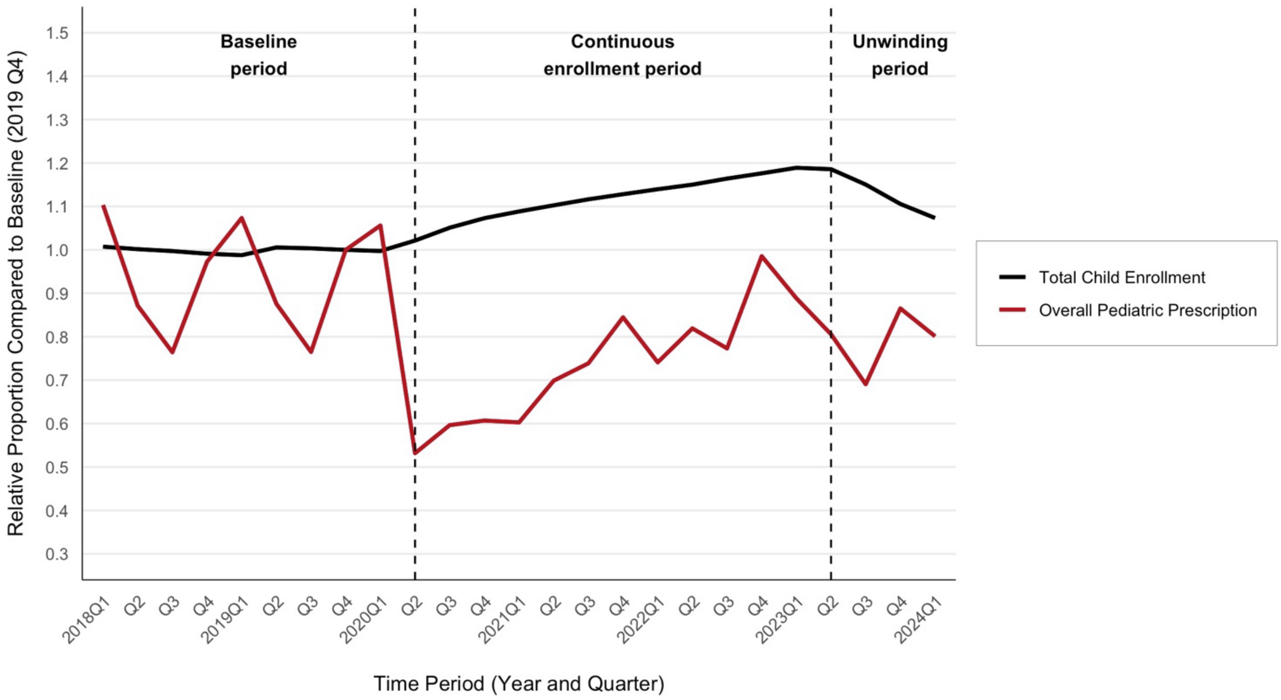

© 2025 Rome BN et al. *JAMA Health Forum*.
