# Supplementary material for: Changes in Medication Use During Medicaid Continuous Enrollment and Unwinding
Source: JAMA Health Forum. 2026 Jan 2;7(1):e255890. doi: 10.1001/jamahealthforum.2025.5890 (PMC12761332; doi:10.1001/jamahealthforum.2025.5890)
Supplement: Supplement 2. — Data Sharing Statement [file jamahealthforum-e255890-s002.pdf]

Data Sharing Statement

Rome. Changes in Medication Use During Medicaid Continuous Enrollment and Unwinding. *JAMA Health Forum*. Published January 02, 2026. doi:10.1001/jamahealthforum.2025.5890

Data
Data available: No

Additional Information
Explanation for why data not available: The Medicaid State Drug Utilization data are publicly available. Medicaid claims were accessed under a Data Use Agreement. Interested parties can reach out to ResDAC to request access at their own expense.
